# *Plasmodium falciparum* histidine-rich protein 2 diversity in Ghana

**DOI:** 10.1186/s12936-020-03328-z

**Published:** 2020-07-16

**Authors:** Otchere Addai-Mensah, Bismarck Dinko, Mark Noagbe, Selassie Louis Ameke, Max Efui Annani-Akollor, Eddie-Williams Owiredu, Kofi Mensah, Richmond Tackie, Eliezer Togbe, Comfort Agyare-Kwabi, Charles Gyasi, Constance Adu-Gyamfi, Alexander Yaw Debrah

**Affiliations:** 1 Department of Medical Diagnostics, Faculty of Allied Health Sciences, Kwame Nkrumah University of Science and Technology, Kumasi, 00233 Ghana; 2 School of Basic and Biomedical Sciences, University of Health and Allied Sciences, Ho, Ghana; 3 Laboratory Department, Ho Municipal Hospital, Ho, Ghana; 4 Department of Molecular Medicine, School of Medicine and Dentistry, Kwame Nkrumah University of Science and Technology, Kumasi, Ghana

**Keywords:** Malaria, *Plasmodium falciparum*, Histidine-rich protein 2, Rapid diagnostic test, Ghana

## Abstract

**Background:**

In the absence of microscopy, *Plasmodium falciparum* histidine-rich proteins 2 (PfHRP2)-based rapid diagnostic tests (RDTs) are recommended for the diagnosis of falciparum malaria, particularly in endemic regions. However, genetic variability of the *pfhrp2* gene threatens the usefulness of the test due to its impact on RDT sensitivity. This study aimed to investigate the diversity of *pfhrp2* in malaria cases among children in Ghana.

**Methods:**

A cross-sectional study was conducted at the Adidome Government Hospital in the Volta Region of Ghana. A total of 50 children with mean age of 6.6 ± 3.5 years and diagnosed falciparum malaria were included. Blood samples were collected for complete blood count, malaria parasite identification and counting using auto analyzer and microscopy, respectively. DNA was isolated from blood-spotted Whatman filters, amplified and sequenced. Nucleotide sequences were translated in silico to corresponding amino acids and the deduced amino acids sequences were analyzed for diversity using Mega X.

**Results:**

The number of repeats and number of each repeat within PfHRP2 varied between isolates. Twelve rare PfHRP2 repeat types, two of which are previously unreported, were identified in this study. The HRP2 sequence obtained in this study shared high similarities with isolates from Kenya. Using Baker’s regression model, Group B was the highest occurring type (58.0%). Screening of all sequences for epitopes recognized by PfHRP2-specific monoclonal antibodies (mAbs), the predominant motif was AHHAADAHH, which is recognized by the C1-13 mAbs.

**Conclusion:**

This study reports diversity of *P. falciparum* HRP2 in samples from Ghanaian children with symptomatic malaria. The findings of this study highlight the existence of extra amino acid repeat types which adds to the PfHRP2 antigenic variability.

## Background

Malaria, which causes substantial morbidity and mortality, is a major public health problem in sub-Saharan Africa, Asia and Latin America [1]. It claims the life of a child under 5 years old every two minutes in sub-Saharan Africa and has annual infection and mortality rates of 191 million and 395,000 individuals, respectively [2, 3]. In Ghana, malaria remains a major cause of loss of days of healthy life, accounting for about 20% of child deaths, 40% of child hospital admissions and more than 50% of outpatient attendances [4–7].

Although microscopic examination of stained blood smears remains the gold standard for malaria diagnosis, its benefit is limited by the absence of adequate skilled personnel and infrastructural and logistic resources, particularly in resource-poor areas [8]. To overcome this problem, the World Health Organization (WHO) included rapid diagnostic tests (RDTs), a less expensive and easily accessible test, as one of the alternative testing systems for malaria diagnosis prior to the prescription of anti-malarial drugs.

Since its development in the 1990s, and with more than 200 currently available brands, there have been consistent reports of observable increase in malaria RDT sales worldwide [9, 10]. Most malaria RDTs exploit the presence of *Plasmodium falciparum* histidine-rich protein 2 (PfHRP2) for the detection of *P. falciparum* [11]. The presence of repetitive epitopes that enable their detection by multiple antibodies and their abundance in blood during the blood-stage of malaria infections has made PfHRP2 a common antigenic target for RDTs [12, 13].

In 2010, Ghana implemented the test-before-treat guideline for malaria where RDT use was promoted to facilitate diagnosis [14]. However, beside low parasite levels especially in asymptomatic cases, improper interpretation of RDT results and/or the handling and storage of RDT kits, deletion of the *pfhrp2* gene and extensive antigen diversity have contributed to discrepancies in RDT sensitivity [15–20], threatening the future use of the test method, particularly in malaria-endemic regions, such as Ghana. Indeed, a recent study in Ghana reported *pfhrp2* gene deletion in 33 and 36% of microscopically-confirmed and PCR-confirmed RDT positive samples, respectively [21]. Over the past decade, several countries, especially in Africa, have reported cases of *P. falciparum* isolates with deleted *pfhrp2*, and isolates with high *pfhrp2* diversity [17, 18, 22–26], with potential negative implications for malaria control and elimination programmes. These notwithstanding, studies on *pfhrp2* gene deletion and diversity of the *pfhrp2* gene in Ghana, a malaria-endemic country, are lacking.

This study aimed to investigate the diversity of PfHRP2 in malaria cases among children in Ghana.

## Methods

### Study design/setting and participants

A cross-sectional study was conducted between January and June 2019 at the Adidome Government Hospital in the Volta Region of Ghana. The Volta Region has perennial malaria transmission, with the predominant parasite being *P. falciparum*. The regional prevalence of falciparum malaria is 45.27% among children [27]. In the study, 50 children between the ages of 3 and 11 years diagnosed with falciparum malaria were included. Samples were obtained prior to initiation of anti-malarial therapy.

### Sample collection and laboratory analysis

Three ml of blood was aseptically obtained from each participant and dispensed into K3 EDTA tubes. Complete blood count was performed on the anticoagulated whole blood using Sysmex KX-21 N auto analyzer (Sysmex Corporation, Japan). To reassess falciparum malaria, thick and thin blood films were prepared and stained with 10% Giemsa for microscopic identification and counting of parasites. The parasite density was calculated by assuming a standard white blood cell (WBC) count of 8000/μl or 4.5 million red blood cells (RBCs)/μl in accordance with WHO standards [28].

Additionally, 5 drops of the blood (each drop corresponding to about 30 μl of blood) were spotted onto Whatman™ filter papers (Schleicher and Schuell BioScience Inc, Keene, New Hampshire, USA), air dried and individually kept in zip-lock plastic bag for subsequent PCR analysis.

### Parasite DNA extraction and molecular analysis

DNA isolation from Whatman filter papers was based on the Chelex-based technique as previously described [29]. PCR amplification of the exon 2 of the *pfhrp2* gene was performed using the semi-nested amplification approach, as previously described by Baker et al. [16]. PCR reactions were carried out in 25 µl volume for the primary and 35 µl volume for the semi-nested reactions. The forward and reverse primers targeting the exon 2 of the *pfhrp2* gene are shown in Additional file 1: Table S1. For both primary and secondary PCR reactions, DNA was denatured at 96 °C for 10 min followed by 40 cycles of denaturation at 95 °C for 50 s, annealing at 55 °C for 50 s, extension at 68 °C for 1 min and a final extension at 72 °C for 5 min. Genomic DNA from 3D7 (wild type) *P. falciparum* and nuclease-free water were used as positive and negative controls, respectively. After secondary amplification, amplicons were separated by electrophoresis on 2% agarose gels, stained with ethidium bromide and visualized under UV light. Sequencing of the *pfhrp2* gene was performed by Inqaba Biotechnical Industries (Pty) Ltd, South Africa (https://www.inqababiotec.co.za/). Nucleic acid sequences were deposited at the National Center for Biotechnology Information (NCBI) (Genbank accession numbers: MT094447-79).

### Data analysis

**Fig. 1:**
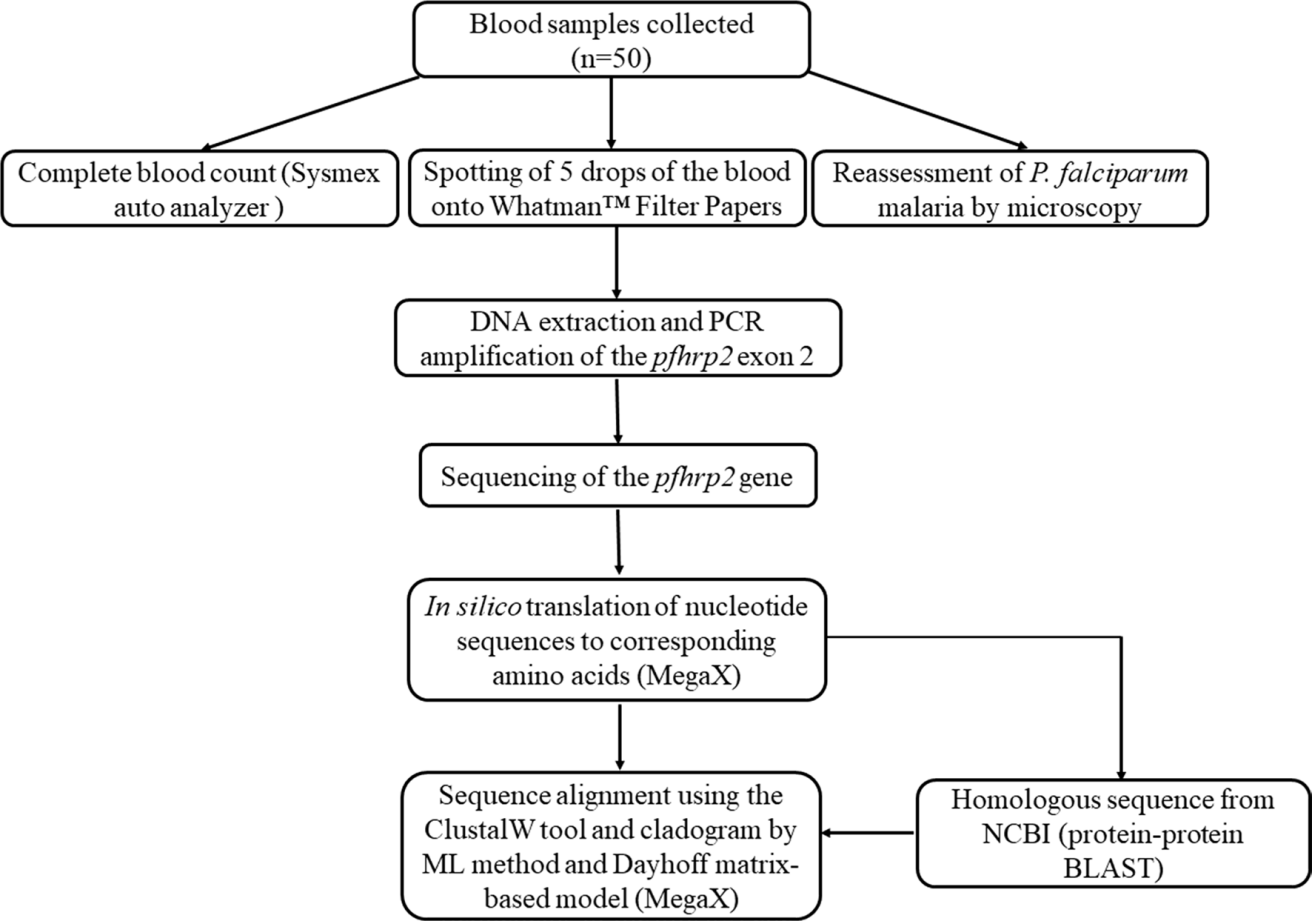
Flow chart of sampling, testing and analysis

Mega X version 10.1.6 [30] was used for sequence analysis. Nucleotide sequences were translated in silico to corresponding amino acids using the correct open reading frame. Amino acid repeats were numerically coded based on previous reports [16, 18, 31]; the frequencies and percentages of each amino acid repeat were estimated. To compare amino acid sequences in this study with previously published data, protein–protein BLAST (BLASTP) analysis of the sequences obtained in this study was performed to obtain homologous sequences the GenBank database at the NCBI. The PfHRP2 amino acid sequences from this study and the homologous sequences downloaded from the NCBI were aligned using the ClustalW tool and a cladogram was built using the Maximum Likelihood method and Dayhoff matrix-based model, with bootstrap consensus tree inferred from 1000 replicates. Although RDTs results were unavailable, the study also sought to determine the diversity of PfHRP2 with respect to RDT sensitivity. To achieve this, the product of repeats type 2 and 7 was calculated; classification and interpretation was based on Baker’s regression model [16]. Screening of the sequences from this study for epitopes recognized by PfHRP2-specific monoclonal antibodies (mAbs) [32] were also performed. Flow chart of sampling, testing and analysis is shown in Fig. 1.

## Results

A total of 50 children with mean age of 6.6 ± 3.5 years were included. There were more females than males (56.0 *vs* 44.0%). The average RBC count, haemoglobin, haematocrit, mean cell volume, mean cell haemoglobin, mean cell haemoglobin concentration, WBC count, platelet count, and parasite density were 4.0 ± 0.7 × 10^6^/µL, 10.5 ± 1.6 g/dL, 30.8 ± 4.6%, 77.2 ± 6.4 fL, 26.7 ± 4.1 pg, 34.5 ± 3.7 g/dL, 7.5 ± 2.7 × 10^3^/µL, 101.0 (63.0–171.3) × 10^3^/µL, and 35,411.0 (15,183.9–65,280.7)/µL, respectively (Table 1).

**Table 1:** Demographics and haematological profile of the study population

Variables	Mean ± SD	Min–max
Age (years)	6.6 ± 3.5	1–14
Gender^a^	n	%
Female	28	56.0
Male	22	44.0
RBC (×10^6^/µL)	4.0 ± 0.7	2.0–5.3
Hb (g/dL)	10.5 ± 1.6	6.3–13.7
HCT (%)	30.8 ± 4.6	16.1–38.6
MCV (fL)	77.2 ± 6.4	62.1–90.8
MCH (pg)	26.7 ± 4.1	18.7–45.6
MCHC (g/dL)	34.5 ± 3.7	29.9–57.8
WBC (×10^3^/µL)	7.5 ± 2.7	2.4–12.8
PLT (×10^3^/µL)^b^	101.0 (63.0–171.3)	20.0–365.0
Parasite density (parasites/µL)^b^	35,411.0 (15,183.9–65,280.7)	568.0–1,033,730.0

*RBC* red blood cell count, *Hb* haemoglobin, *HCT* haematocrit, *MCV* mean cell volume, *MCH* mean cell haemoglobin, *MCHC* mean cell haemoglobin concentration, *WBC* white blood cell count, *PLT* platelet count

^a^Presented as frequencies and percentages

^b^Presented as medians (interquartile ranges)

Thirty-three different PfHRP2 amino acid sequences were identified among 50 PfHRP2 sequences obtained in this study. The size of PfHRP2 ranged from 225 to 304 amino acids among all isolates and 25 to 38 amino acid repeat types per isolate. The total number of repeats and the number of each repeat within PfHRP2 varied between isolates. Repeat types 2, 6, 7 and 8 were observed in 100% of the isolates. Repeat types 5 and 12 were observed in 98% whereas types 1, 3 and 10 were found in 92–96% of the samples. The repeat types 4 and 13 occurred in 26 and 10% of the isolates, respectively. None of the samples had repeat types 9 and 11 (Table 2).

**Table 2:** Frequencies and proportions of pfhrp2 amino acid repeat types

Code	Repeat sequences	Repeat frequency	Repeat percentage	Range
Type 1 motif	AHHAHHVAD	142	94.0	0–9
Type 2 motif	AHHAHHAAD	572	100.0	6–16
Type 3 motif	AHHAHHAAY	67	92.0	0–3
Type 4 motif	AHH	20	26.0	0–3
Type 5 motif	AHHAHHASD	58	98.0	0–3
Type 6 motif	AHHATD	178	100.0	2–7
Type 7 motif	AHHAAD	312	100.0	3–12
Type 8 motif	AHHAAY	65	100.0	1–3
Type 10 motif	AHHAAAHHATD	74	96.0	0–2
Type 12 motif	AHHAAAHHEAATH	50	98.0	0–1
Type 13 motif	AHHASD	5	10.0	0–1

Twelve rare PfHRP2 repeat types, two of which are previously unreported, were identified in this study; the two were A**PD**AHHVAD and AHHAAAH**D**EAA**LI**. Of the rare repeat types, types 2 (AHHAHHAA**H**) and 7 **(**AHHAA**H**) had the highest frequency (Table 3).

**Table 3:** List of rare and novel pfhrp2 amino acid repeat types identified in Ghana

Code	Known repeats	Rare/novel repeats	Repeat frequency	Repeat percentage
Type 1 motif	AHHAHHVAD	AHHAHHVA**Y**	3	6.0
		AHH**T**HHVAD	1	2.0
		A**PD**AHHVAD^a^	1	2.0
Type 2 motif	AHHAHHAAD	AHHAHHA**P**D	1	2.0
		AHHAHHA**D**D	2	4.0
		AHHAHHAA**H**^b^	5	10.0
Type 5 motif	AHHAHHASD	AHHA**P**HASD	1	2.0
Type 7 motif	AHHAAD	AHHAA**H**^b^	6	12.0
		AHHA**D**D	1	2.0
Type 10 motif	AHHAAAHHATD	AHHAA**T**HHATD	1	2.0
Type 12 motif	AHHAAAHHEAATH	AHHAAAHHEAA**S**H	3	6.0
		AHHAAAH**D**EAA**LI**^a^	1	2.0

Boldened amino acid shows the position where the rare/novel repeat types vary compared to known repeat types

^a^Previously unidentified repeat

^b^More than one repeat copies per isolate

**Fig. 2:**
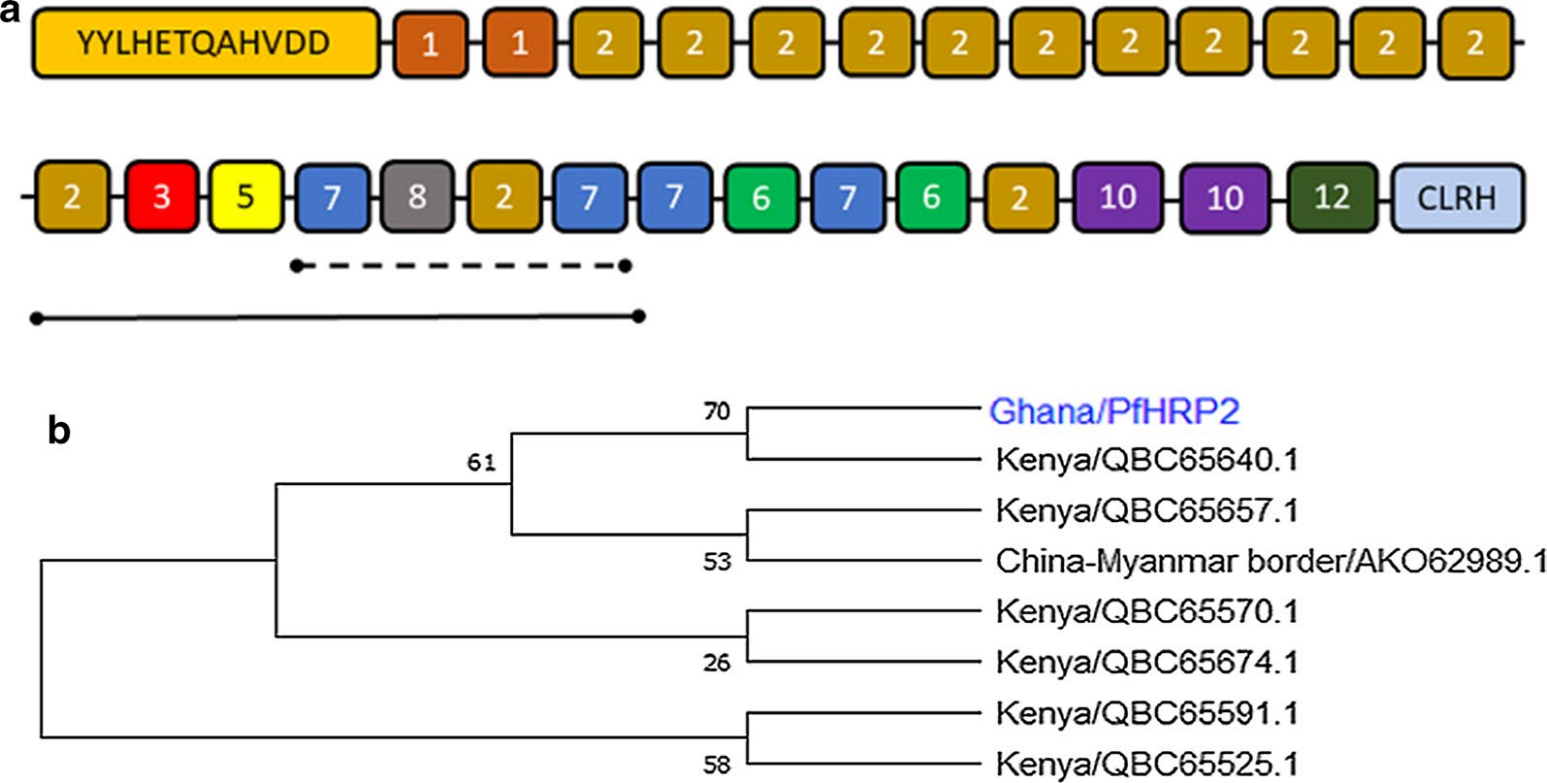
Structural organization and relatedness between the Ghanaian pfhrp2 sequences and homologous sequences from other regions. **a** Schematic diagram of the structural organization of pfhrp2 amino acid repeat types in Ghana. The solid line represents semi-conserved amino acid repeat motif. The short dashes represent partial amino acid repeat motif. **b** Cladogram showing the relatedness between the Ghanaian pfhrp2 sequences and homologous sequences from other regions

Predominant repeat types in this study were used to model the structural organization of PfHRP2 in Ghana. Although the structural organization of the PfHRP2 repeat types was variable, the repetitive regions found in most of the samples from this study started with type 1 (94.0%) and all PfHRP2 sequences terminated with type 12 (100.0%) (Fig. 2a). Fifty-four per cent of the isolates had a semi-conserved PfHRP2 repeat type motif composed of repeat types 2, 3, 5, 7, 8, 2, and 7. Partial amino acid repeat motif comprising types 7, 8, 2 and 7 was found in 34.0% of the isolates.

To explore the similarities between the modelled PfHRP2 sequence obtained from the Ghanaian isolates and those from other regions available at the NCBI, BLASTP analysis of the amino acid sequences were performed. Seven hits (Accession numbers: QBC65525.1, QBC65570.1, QBC65591.1, QBC65640.1, QBC65657.1, QBC65674.1 from isolates in Kenya; AKO62989.1 from China–Myanmar border area) were obtained (Fig. 2b; Additional file 1: Table S2). The modelled HRP2 sequence shared 85–94% similarity with Kenyan isolates and 94% similarity with the isolates from China–Myanmar border area. BLASTP of the sequences from each of the 50 samples revealed that 78.0% (39/50) have high similarities with isolates from Kenya, highlighting possible shared identity between PfHRP2 from Ghana and Kenya (Additional file 1: Table S3).

Although data on RDTs were unavailable, an obvious limitation of the study, the Baker model [16] was used to determine the distribution on the basis of PfHRP2 diversity with respect to RDT sensitivity. Isolates were classified as Groups A, B, I and C if their Baker repeat (type 2 × type 7) was > 100, 50–100, 44–49 and < 43, respectively. Group B was the highest occurring type (58.0%), followed by Group C (22.0%) (Table 4).

**Table 4:** Frequency of occurrence of pfhrp2 groups (Baker model) in Ghana

Diversity	Frequency	Percentages
A	2	4.0
B	29	58.0
C	11	22.0
I	8	16.0

Due to the relatively high percentage of Group C isolates (22.0%), which have been reported to be associated with RDT non-sensitivity [33] obtained in this study, the distribution of possible epitopes to be targeted by mAb RDTs based on the study by Lee et al. [32] was explored. The predominant motif among the 50 isolates was AHHAADAHH, which is recognized by the C1-13 mAb, followed by AHHAHHA, recognized by mAb 3A4. None of the isolates in this study had the AYAHHAHHAAY motif, while the HAHHAHHAADAHH motif, recognized by C2-3, occurred at a lower frequency (Table 5).

**Table 5:** Distribution of potential epitopes targeted by monoclonal antibodies in rapid diagnostic tests among Ghanaian isolates

Monoclonal antibody	Epitope motif	Samples containing motif [n (%)]	Motif per isolate
C1-13	AHHAADAHH	50 (100)	12–21
3A4	AHHAHHA	50 (100.0)	11–18
N7	DAHHAADAHHA	50 (100)	3–8
2G12-1C12	DAHHAADAHH	50 (100)	2–8
A6-4	HATDAHH	50 (100)	3–6
PTL-3	YAHHAHHA	50 (100)	1–4
S2-5	AHHASDAHHA	48 (96.0)	0–3
1E1-A9	AHHAHHV	47 (94.0)	0–9
TC-10	TDAHHAADAHHAADA	42 (84.0)	0–2
C2-3	HAHHAHHAADAHH	16 (32.0)	0–2
Genway	AYAHHAHHAAY	0 (0.0)	0

## Discussion

In Africa, *P. falciparum* is the most common malaria-causing parasite. Microscopy is the gold standard for the diagnosis of malaria; however, in its absence, RDT has been recommended by WHO for use in malaria diagnosis. The majority of commercially available RDTs target the PfHRP2; however, the future benefit of these RDTs is in jeopardy due to reports of *P. falciparum* isolates that lack the *pfhrp2* gene (deletion) and the presence of isolates with variants of the gene (diversity) [15–19, 22, 23]. It is thus of public health significance to assess the diversity of the *pfhrp2*, especially in different parts of Africa where the disease exerts a high rate of morbidity and mortality, particularly among children.

This study presents an analysis of the diversity of *pfhrp2* among *P. falciparum* isolates from children in Ghana. In contrast with a study by Amoah et al. in Ghana, who reported *pfhrp2* gene deletion in 33 and 36% of microscopically confirmed and PCR-confirmed RDT-positive samples, respectively [21], all the isolates from this study achieved successful sequencing of the *pfhrp2* gene, indicating absence of deletion. This finding is in harmony with studies by Baker et al. [16, 20] and Nderu et al. [18]. Compared with the study by Amoah et al., which was conducted in coastal parts of Ghana, the lack of *pfhrp2* gene deletion in this study (conducted in the middle belt) suggests that geographical differences could play a role in *pfhrp2* gene diversity. Studies in other regions of the country are thus warranted. It should be noted, however, that inclusion of only symptomatic malaria cases and the limited sample size may have influenced the findings of this study. A larger sample size is recommended in future studies.

After in silico translation of the *pfhrp2* genes into amino acid sequences, the isolates from this study shared some characteristics with previous published data. Amino acid repeat types 2, 6, 7 and 8 were found in all isolates whereas types 9 and 11 were absent. Few of the isolates had the type 4 repeat and only 10% had the type 13. All other repeat types were found in over 90% of the isolates. These findings are consistent with previous reports from other countries [18, 20, 31, 33–35].

The structural organization of the amino acid repeat types found in this study was highly diverse. Out of the 33 unqiue PfHRP2 sequences obtained, only a third occurred more than once. However, some repeat organizations were shared between isolates. Most of the sequences started with type 1 and all terminated with type 12 as is consistent with previous studies [18–20, 31]. Additionally, a semi-conserved PfHRP2 repeat type motif (types 2, 3, 5, 7, 8, 2, 7) and partial repeat motif (types 7, 8, 2, 7) were found in about half and a quarter of the isolates, respectively. This is similar to the findings of Baker et al. [20] and Nderu et al. [18]. Phylogenetic analysis of the Ghana PfHRP2 revealed striking similarities with isolates from Kenya. In a study along the Chinese-Myanmar border, novel PfHRP2 repeat types arising from replacement of a single amino acid of eight amino acid repeats types were identified [36]. Nderu et al. [18] also found 39 novel PfHRP2 repeat types. In India, Bharti et al. reported five novel repeat types [19]. In this study, two novel PfHRP2 repeat types (A**PD**AHHVAD and AHHAAAH**D**EAA**LI**) were identified in addition to 10 of the recently reported novel repeats which occurred at low frequencies. Together with previous reports, the findings of this study support the presence of yet-to-be defined repeat types and highlights that these novel types occur at relatively lower frequencies.

PfHRP2 diversity has been reported to influence the diagnosis of malaria using PfHRP2-based RDTs. In 2005, Baker et al. demonstrated, using logistic regression model, that the product of repeat types 2 and 7 affect inter-study sensitivity variation of PfHRP2-based RDTs, especially for samples with parasite densities ≤ 250 parasites/μl [16]. In 2010 however, it was found, using isolates from different geographical areas, that type 2 × type 7 was not associated with RDT sensitivity [20]. Nonetheless, subsequent studies by Kumar et al. [33] in 2012 and Wurtz et al. [37] in 2013 observed an association between the Group C category (type 2 × 7 < 43) and RDT false negativity and reduced limit of detection. Most of the isolates in this study were Group B (type 2 × 7 = 50–100) as consistent with previous studies in Africa [18, 38, 39]. To investigate the distribution of possible epitopes to be targeted by monoclonal antibodies in RDTs, a search for the 11 common epitopes recognized by commercially available mAb [32] among the isolates was performed. The most occurring epitope was AHHAADAHH, recognized by the C1-13 mAb, followed by AHHAHHA, recognized by mAb 3A4. The AYAHHAHHAAY motif was not detected in this study and HAHHAHHAADAHH occurred at a lower frequency. This is consistent with recent reports by Fontecha et al. [31] and Willie et al. [40] and corroborates with an earlier report by Lee et al. that RDTs which employ the C2-3 and Genway mAbs are less sensitive [32].

It is worthy of note that, although deletion/diversity of *pfhrp2* gene could potentially impact RDT sensitivity, the product of the *pfhrp3* gene can compensate for its absence in relation to the sensitivity of the tests. Moreover, RDTs recommended by WHO are capable of detecting the PfHRP2/PfHRP3 antigens together with parasite-specific lactate dehydrogenase (pLDH) or *Plasmodium* aldolase [11]. Thus, the absence of the PfHRP2 antigen (due to gene deletion/diversity) does not invalidate the available RDTs that are based on the other markers of infection. Furthermore, a major limitation of the study, which is worth considering by future studies in the region, is the unavailability of data on RDT.

## Conclusion

This study reports diversity of *P. falciparum* histidine-rich proteins 2 in samples from Ghanaian children with symptomatic malaria. Additionally, the findings of this study highlight the existence of extra amino acid repeat types which adds to the PfHRP2 antigenic variability. The findings of this study will contribute to the understanding of the performance of PfHRP2-based RDTs in the Ghanaian setting.

## Supplementary information

**Additional file 1.** Primers used, BLASTP hits for HRP2 sequence modelled from Ghanaian isolates and BLASTP of Ghanaian HRP2 sequences for all 50 samples.

## Data Availability

The datasets supporting the conclusions of this article are included within the article and its additional file.

## Abbreviations

PfHRP2: *Plasmodium falciparum* histidine-rich proteins 2
WHO: World Health Organization
RDT: Rapid diagnostic test
PCR: Polymerase chain reaction
BLAST: Basic Local Alignment Search Tool
mAb: Monoclonal antibodies
