# Supplementary material for: Plasmodium falciparum histidine-rich protein 2 diversity in Ghana
Source: Malar J. 2020 Jul 16;19:256. doi: 10.1186/s12936-020-03328-z (PMC7364488; doi:10.1186/s12936-020-03328-z)
Supplement: Supplementary file 1 — Additional file 1. Primers used, BLASTP hits for HRP2 sequence modelled from Ghanaian isolates and BLASTP of Ghanaian HRP2 sequences for all 50 samples. [file 12936_2020_3328_MOESM1_ESM.docx]

***Plasmodium falciparum* histidine-rich protein 2 diversity in Ghana**

Otchere Addai-Mensah^1,*^, Bismarck Dinko^2^, Mark Noagbe^1^, Selassie Louis Ameke^3^, Max Efui Annani-Akollor^4^, Eddie-Williams Owiredu^4^, Kofi Mensah^1^, Richmond Tackie^2^, Eliezer Togbe^1^, Comfort Agyare-Kwabi^2^, Charles Gyasi^1^, Constance Adu-Gyamfi^1^, Alexander Yaw Debrah^1^

**Table S1. Primers used**

| **Primer** | **Sequence** |
| --- | --- |
| PfHRP2-Forward 1 | 5´-CAAAAGGACTTAATTTAAATAAGAG-3´ |
| PfHRP2-Reverse 1 | 5´-AATAAATTTAATGGCGTAGGCA-3´ |
| PfHRP2-Forward 2 | 5’- ATTATTACACGAAACTCAAGCAC-3’ |

**Table S2. BLASTP hits for HRP2 sequence modelled from Ghanaian isolates**

| **Accession numbers** | **% identity** | **E value** |
| --- | --- | --- |
| QBC 65640.1 | 93.02 | 1.00E-90 |
| QBC65570.1 | 85.22 | 1.00E-90 |
| QBC65657.1 | 93.82 | 1.00E-89 |
| QBC65591.1 | 93.33 | 4.00E-89 |
| QBC65674.1 | 94.21 | 2.00E-86 |
| QBC65525.1 | 93.44 | 1.00E-85 |
| AKO62989.1 | 94.33 | 3.00E-82 |

**Table S3. BLASTP of Ghanaian HRP2 sequences for all 50 samples.**

| **Sample ID** | **E value** | **Country** | **Accession number*** |
| --- | --- | --- | --- |
| Sample 1 | 5.00E-98 | Uganda | AUG44600.1 |
| Sample 2 | 6.00E-105 | Kenya | QBC65733.1 |
| Sample 3 | 2.00E-117 | Kenya | QBC65732.1 |
| Sample 4 | 7.00E-84 | India | AQY62062.1 |
| Sample 5 | 2.00E-86 | Kenya | QBC65756.1 |
| Sample 6 | 1.00E-92 | Kenya | QBC65697.1 |
| Sample 7 | 7.00E-84 | India | AQY62062.1 |
| Sample 8 | 8.00E-89 | Central America | QBC25308.1 |
| Sample 9 | 1.00E-100 | Kenya | QBC65698.1 |
| Sample 10 | 8.00E-89 | Central America | QBC25308.1 |
| Sample 11 | 2.00E-86 | Kenya | QBC65756.1 |
| Sample 12 | 5.00E-96 | Kenya | QBC65754.1 |
| Sample 13 | 3.00E-99 | Kenya | QBC65755.1 |
| Sample 14 | 9.00E-89 | Central America | QBC25307.1 |
| Sample 15 | 3.00E-99 | Kenya | QBC65755.1 |
| Sample 16 | 4.00E-92 | Kenya | QBC65751.1 |
| Sample 17 | 4.00E-92 | Kenya | QBC65751.1 |
| Sample 18 | 7.00E-114 | Madagascar | APD77410.1 |
| Sample 19 | 3.00E-91 | Kenya | QBC65721.1 |
| Sample 20 | 3.00E-108 | Papua New Guinea | AUB13318.1 |
| Sample 21 | 9.00E-89 | Central America | QBC25307.1 |
| Sample 22 | 3.00E-91 | Kenya | QBC65721.1 |
| Sample 23 | 3.00E-99 | Kenya | QBC65755.1 |
| Sample 24 | 5.00E-98 | Uganda | AUG44600.1 |
| Sample 25 | 3.00E-91 | Kenya | QBC65721.1 |
| Sample 26 | 5.00E-96 | Kenya | QBC65754.1 |
| Sample 27 | 3.00E-91 | Kenya | QBC65753.1 |
| Sample 28 | 5.00E-96 | Kenya | QBC65754.1 |
| Sample 29 | 7.00E-108 | Kenya | QBC65731.1 |
| Sample 30 | 6.00E-105 | Kenya | QBC65733.1 |
| Sample 31 | 5.00E-98 | Uganda | AUG44600.1 |
| Sample 32 | 7.00E-93 | Kenya | QBC65736.1 |
| Sample 33 | 1.00E-104 | Kenya | QBC65738.1 |
| Sample 34 | 5.00E-102 | Kenya | QBC65739.1 |
| Sample 35 | 6.00E-98 | Kenya | QBC65741.1 |
| Sample 36 | 6.00E-92 | Kenya | QBC65700.1 |
| Sample 37 | 6.00E-80 | Kenya | QBC65701.1 |
| Sample 38 | 2.00E-86 | Kenya | QBC65756.1 |
| Sample 39 | 5.00E-96 | Kenya | QBC65754.1 |
| Sample 40 | 3.00E-93 | Kenya | QBC65714.1 |
| Sample 41 | 1.00E-99 | Kenya | QBC65746.1 |
| Sample 42 | 5.00E-90 | Kenya | QBC65660.1 |
| Sample 43 | 1.00E-110 | Kenya | QBC65661.1 |
| Sample 44 | 9.00E-103 | Kenya | QBC65662.1 |
| Sample 45 | 2.00E-101 | Kenya | QBC65663.1 |
| Sample 46 | 6.00E-108 | Kenya | QBC65664.1 |
| Sample 47 | 7.00E-93 | Kenya | QBC65736.1 |
| Sample 48 | 2.00E-112 | Kenya | QBC65665.1 |
| Sample 49 | 1.00E-92 | Kenya | QBC65703.1 |
| Sample 50 | 4.00E-83 | Kenya | QBC65728.1 |

*Accession numbers presented are those with the highest percentage identity.
